# Association of the dietary inflammation index with frailty in middle-aged and older adults: a systematic review and meta-analysis

**DOI:** 10.3389/fnut.2025.1607110

**Published:** 2025-07-02

**Authors:** Shu-min Zhu, Ping Chang, Zhen Wang, Bei Yang, Hong-fang Ye

**Affiliations:** ^1^Department of Nursing, Nanjing Drum Tower Hospital, Affiliated Hospital of Medical School, Nanjing University, Nanjing, China; ^2^School of Nursing, Nanjing University of Chinese Medicine, Nanjing, China

**Keywords:** dietary inflammation index, frailty, inflammations, diet, middle-aged and older adults, meta-analysis

## Abstract

**Background:**

Low-grade of chronic inflammation is a signature of the aging and physiologic frailty may be related to a dysfunctional homeostasis between pro- and anti-inflammatory systems mediated by diverse determinants, including dietary constituents that produce a wide range of biologically active substances, which are important modulators of inflammation in the organism. The Dietary Inflammation Index (DII), a quantitative measure of diet-associated inflammation, has been widely used in studies of a variety of chronic inflammation-related diseases, but the correlation between the DII and frailty has not been uniformly determined.

**Method:**

We searched multiple databases, including CNKI, Wanfang, VIP, Chinese Biomedical Literature Database, PubMed, Embase, Scopus, and Web of Science, to identify studies in English and Chinese examining the association between the dietary inflammatory index and frailty risk. Literature was searched from the time of database construction to January 2025. Two standardized scales were employed for quality assessment: NOS for longitudinal studies and AHRQ tools for cross-sectional research. Sensitivity analyses and publication bias tests were performed using Stata 15.0, and meta-analyses were performed using RevMan 5.3 to calculate the combined odds ratio (OR) and its 95% confidence interval (CI) to assess the DII in correlation with pre-frailty and frailty.

**Results:**

The meta-analysis examined 15 studies involving a total of 42,130 study participants. The combined results showed that individuals were at increased risk of both frailty [OR = 1.47, 95%CI (1.28, 1.69), *p* < 0.001] and pre-frailty [OR = 1.54, 95%CI (1.34, 1.76), *p* < 0.001] in the highest DII category compared to the lowest DII category. Subgroup analyses revealed that DII was significantly and positively associated with the increased risk of frailty in all subgroups of different study geographic areas, types, sample sizes, and dietary assessment tools, whereas the difference between frailty occurrence assessed using the FI debilitation index and DII was not statistically significant in the subgroups of different debilitation assessment tools.

**Conclusions:**

Available evidence suggests that high pro-inflammatory diets may be associated with an increased risk of frailty, and that dietary strategies that lower the DII could play a role in reducing frailty incidence among older and middle-aged groups.

## 1 Introduction

Frailty is a complex age-related clinical syndrome marked by diminished physiological reserves, reduced stress resistance, and heightened vulnerability in aging individuals (1). It is closely related to the occurrence of various serious diseases including but not limited to cardiovascular disorders, depressive symptoms, renal dysfunction, and malignancies, which may precipitate multiple negative health consequences including elevated fall susceptibility, diminished quality of life, higher hospitalization frequency, and increased mortality risk (2, 3). Although frailty has traditionally been thought to affect mainly the older adult population (≥65 years of age), there is growing evidence that the pathologic process may begin in midlife (40–65 years of age), with a pre-frailty state of decreased strength, increased fatigue, and metabolic abnormalities, and progress to the typical frailty of old age through the mechanism of “cumulative deficits” (4, 5). The study emphasizes that this situation is not static; it is actually a state that can change with intervention. By addressing modifiable risk factors, we have the potential to reverse its course (6, 7).

Elevated levels of pro-inflammatory cytokines (e.g., IL-6, TNF-α, and CRP) are prevalent in patients with frailty, and this persistent low-grade inflammatory state accelerates muscle proteolysis and inhibits synthesis, leading to sarcopenia and metabolic dysfunction, which further induces oxidative stress and immune dysregulation and thus exacerbates the symptoms of frailty (8–11). Frailty is strongly associated with chronic low-grade inflammation, and diet is a key modifiable factor in the inflammatory response (12). Studies have shown that pro-inflammatory diets activate the NF-κB pathway and NLRP3 inflammatory vesicles, increase the release of inflammatory factors such as TNF-α and IL-6, and increase the risk of frailty by accelerating muscle loss and metabolic disorders through mechanisms such as chronic low-grade inflammation, oxidative stress, and insulin resistance (13). On the contrary, anti-inflammatory diets rich in polyphenols, omega-3 fatty acids, and vitamin D directly scavenge free radicals and inhibit the NF-κB and MAPK signaling pathways while regulating the intestinal flora, reducing the entry of endotoxin (LPS) into the bloodstream, decreasing the level of systemic inflammation, and delaying the progression of frailty (14). From a multidimensional perspective, frailty involves not only physical functioning, but may also affect a number of psychological, cognitive, and social domains. The present study focuses on physical frailty because it has the clearest assessment criteria and is more well-studied in terms of the mechanisms associated with dietary inflammation.

While the current study demonstrated similar significance in exploring the relationship between dietary scores and frailty (15), the pathophysiological associations with frailty were different: the Geriatric Nutrition Risk Index (GNRI) focused on nutritional adequacy (16); the Healthy Eating Index (HEI) emphasized a different balance of food groups (17); the Mediterranean diet (MDS) was associated with sarcopenia and the prevention of chronic disease (18); and the DASH score focused on improving blood pressure, Lipid levels (19). The specificity of the Dietary Inflammatory Index (DII) is reflected in its unique dimension of inflammation regulation, a tool developed based on global surveillance data to quantitatively assess the potential impact of individual diets on the overall inflammatory response and to reflect the correlation between diet and inflammation (20, 21). DII classifies diets along a spectrum ranging from highly pro-inflammatory to strongly anti-inflammatory, assessing the cumulative impact of various food components on systemic inflammation (20, 22).

Studies have been conducted to assess the correlation between DII and the risk of developing frailty, and while some studies have reported a positive correlation between DII and the risk of frailty (23, 24), others have shown that the two are not significantly correlated (25). Therefore, this study used meta-analysis to synthesize the results of existing studies, and meta-regression and subgroup analyses based on study design, geographic location, and so on, to address the differences in effect sizes due to methodological heterogeneity. The present study aimed to comprehensively characterize the complex relationship between DII and the occurrence of frailty in middle-aged and older adults, thereby laying the foundation for future intervention strategies.

## 2 Method

The present systematic review and meta-analysis followed the PRISMA guidelines (26) and is registered on PROSPERO (CRD42024623860).

### 2.1 Retrieval strategy

A combination of subject terms and free terms was used to fully search PubMed, Embase, Web of Science, Scopus, China National Knowledge Infrastructure, Wanfang Database, VIP Database, and China Biomedical Literature Database for dietary inflammation index and frailty correlation literature, with a timeframe from the construction of the database to January 2025. Use the following search terms to search: (“frailty” OR “frailty syndrome” OR “frail^*^” OR “frailty” OR “debilit^*^” OR “weakness^*^”) AND (“dietary inflammatory index” OR “inflammatory potential of diet” OR “anti inflammatory diet” OR “pro inflammatory diet”).

### 2.2 Inclusion and exclusion criteria

Inclusion criteria covered: (i) research assessing the relationship of Dietary Inflammatory Index scores with frailty susceptibility; (ii)middle-aged and older adults aged ≥45 years; and (iii) data on the correlation between DII and risk of frailty were reported in the literature and effect estimates were provided in the form of OR or HRs and their 95% CI; (iv) study design was either observational (case-control studies, cohort studies, and cross-sectional studies) or randomized controlled studies.

Exclusion criteria included: (i) literature for which full text was not available or duplicate publications; (ii) literature for which relevant outcome indicators were not reported or relevant data (OR or HR) could not be extracted; (iii)original studies that were not published in English or Chinese; (iv) reviews, dissertations, systematic reviews, conference abstracts, book chapters, and patents; (v)Lower quality literature.

### 2.3 Literature screening and data extraction

Two authors independently screened studies based on inclusion/exclusion criteria, consulting a third researcher when necessary to resolve disagreements. Data collection covered three domains: study identification details (author, year, country), methodological elements (study design, population, follow-up, assessment tools, covariates), and analytical outcomes (adjusted risk estimates with confidence intervals)

### 2.4 Literature quality assessment

Methodological quality was systematically evaluated using discipline-specific tools: cohort and case-control studies were appraised with the Newcastle-Ottawa Scale (NOS), where studies achieving scores above 5 out of 9 were deemed methodologically sound (27). Cross-sectional studies were evaluated according to the Agency for Healthcare Research and Quality (AHRQ) criteria, employing an 11-item checklist with binary scoring (1 = present/0 = absent or unclear). Based on cumulative scores, cross-sectional studies were categorized into three quality tiers: low: 0–3, moderate: 4–7 and high: 8–11 quality evidence (28).

### 2.5 Statistical analytics

RevMan 5.3 and Stata 15.0 software were used for statistical processing. Adjusted dominance ratios (ORs) were combined using an inverse variance weighting approach (treating risk ratios (HRs) as OR equivalents), and effect sizes were combined using the model with the highest number of covariates provided by the authors. Study heterogeneity was examined using the χ^2^ statistic, and when there was no significant heterogeneity (*I*^2^ < 50%, *P* > 0.10), a fixed-effects model was used; otherwise (*I*^2^ ≥ 50% or *P* ≤ 0.10), random-effects models were used to combine effect sizes, and subgroup analyses and meta-regressions were performed to explore sources of heterogeneity based on study geographic region, type of study, sample size, and DII assessment tool and frailty assessment tool. Sensitivity analyses were conducted through sequential exclusion of individual studies to evaluate result stability. Publication bias was assessed visually via funnel plots and quantitatively through Egger's and Begg's tests, with trim-and-fill adjustment implemented when required.

## 3 Results

### 3.1 Search results and study characteristics

**Figure 1:**
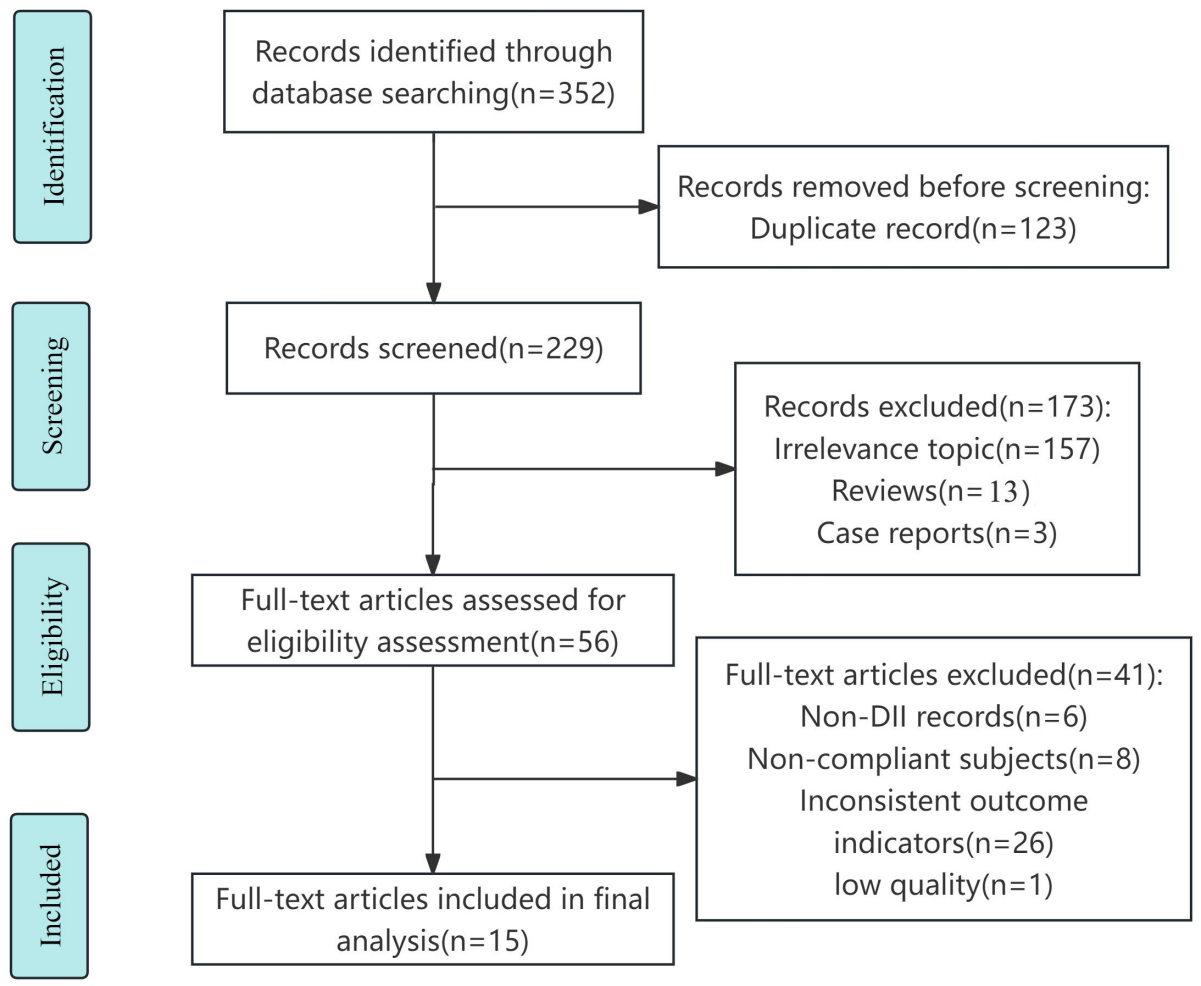
Literature screening process.

The detailed process of literature search and study selection is shown in the flow chart (Figure 1). A total of 352 papers were obtained through database search, and 229 papers were obtained after removing duplicates. After preliminary screening of the papers by checking and reading the titles and abstracts, excluding 157 studies with irrelevant topics, 13 reviews and 3 conference abstracts, and eliminating 26 papers with different endpoints or exposures, 8 papers with incompatible study subjects and 6 papers with non-DII records, as well as 1 low-quality paper through full-text reading, 15 papers (23–25, 29–40) were finally included, of which 12 were in English (23–25, 32–40), 3 were in Chinese (29–31).

### 3.2 Research characteristics

Fifteen (23–25, 29–40) studies were published from 2017 to 2025 involving 42,130 subjects, including nine (25, 29, 30, 32–34, 36, 37, 40) cross-sectional studies, five (24, 25, 35, 38, 39) cohort studies, and one (31)case-control study. DII calculations were all performed using Shivappa's updated scoring system (20), with fourteen (23–25, 29–31, 33–40) reporting outcome indicators in the form of categorical DII, and 6 (23, 25, 31, 36, 37, 40) reporting indicators in continuous DII. Three (25, 29, 36) studies in the literature reported on the risk of developing a pre- frailty stage. Fifteen studies all controlled for the effect of confounding factors on the outcome by means of multifactorial analyses. Quality assessment revealed scores from 6 to 9 across the studies, reflecting a consistently high standard of methodological rigor. Detailed information is shown in the Table 1.

**Table 1:** Basic characteristics and quality evaluation of the retained literature.

**Author, year**	**Country**	**Study design**	**Population**	**Duration (year)**	**Sample size (% female)**	**Age**	**Assessment of DII**	**Assessment of frailty**	**Adjusted confounders**	**Quality score**
ZhaoHui (2023)	China	Cross-sectional	Rural residents	**—**	1,682 (51.66)	66 ± 4	FFQ	FI	Sex, ethnicity, marital status, physical activity	8
Liyi (2023)	China	Cross-sectional	Hospitalized patients	**—**	162 (26.54)	78.01	FFQ	Fried	BMI, literacy nutritional risk assessment, energy intake, protein intake, carbohydrates, polyunsaturated fatty acids	8
Liu Lin (2025)	China	Case-control	Hospitalized patients	**—**	T:118 (43.22) C:120 (51.67)	≥60	FFQ	Fried	Age, comorbidities, lower red blood cell levels, hemoglobin level, total protein level, albumin level	8
Çalapkoru (2024)	Turkish	Cross-sectional	Outpatient visits	**—**	187 (64.00)	70.83 ± 4.98	24-h dietary records	FRAIL	Sex, BMI, nutritional risk assessment, instrumental activity, waist circumference, hip circumference, calf circumference	8
Jung (2024)	Korea	Cross-sectional	Community residents	**—**	950(51.68)	70–84	24-h dietary records	Fried	Sex, age, household Income, literacy, family Structure, smoking status, alcohol consumption status, physical activity, energy intake, number of prescription medications, chewing status, number of chronic conditions	9
Kim (2018)	Korea	Cross-sectional	Community residents	**—**	321 (67.91)	70–85	24-h dietary records	Fried	Sex, energy intake, chewing status	8
Laclaustra (2020)	Spanish	Cohort	Community residents	3	1,915 (51.50)	68.4 ± 6.2	24-h dietary records	Fried	Sex, age, BMI, literacy, smoking status, physical activity, energy intake, disease status	6
LiShuyi (2024)	China	Cohort	Community residents	4	3,035 (50.10)	71.8 ± 4.8	FFQ	Fried	Sex, age, BMI, household Income, living alone, smoking status, physical activity, energy intake, protein intake, subjective social status, cognitive impairment, cancer, diabetes disease, cardiovascular disease	7
LiXiaoxia (2024)	China	Cross-sectional	Community residents	**—**	6,414 (35.74)	≥60	FFQ	FI	Sex, age, household Income, smoking status, alcohol consumption status, energy intake, dietary supplement intake	8
Resciniti (2019)	USA	Cross-sectional	NHANES	**—**	7,182 (52.58)	≥60	24-h dietary records	Fried	Sex, age, race, Literacy, smoking status, comorbidities	7
Shi (2023)	USA	Cross-sectional	NHANES	**—**	1,586 (46.85)	69.42	FFQ	Searle index	Sex, age, race, BMI, household Income, physical activity, alanine aminotransferase, triglycerides, low-density lipoprotein, diabetes disease, hypertension	7
Shivappa (2018)	USA	Cohort	OAI	8	4,421 (58.00)	61.30 ± 9.30	FFQ	SOF index	Sex, age, race, BMI, household Income, literacy, depression, smoking status, physical activity, comorbidities	7
Son (2024)	Japan	Cohort	Community residents	7	811 (47.30)	73.7 ± 4.80	FFQ	Fried	Sex, age, literacy, depression, physical activity, comorbidities, oral health assessment	7
Zaslavsky (2017)	USA	Cohort	Outpatient visits	12	1,0431(100)	50–79	FFQ	Fried	Sex, race, household Income, literacy, smoking status, comorbidities	7
Lin (2024)	USA	Cross-sectional	NHANES	—	2,795 (45.18)	69.76 ± 6.70	24-h dietary records	Searle index	Sex, ethnicity, race, literacy, BMI, smoking status, alcohol consumption status, energy intake, hypertension, cardiovascular disease, duration of the disease, glycosylated hemoglobin	7

T, case group; C, control group; OAI, Osteoarthritis Initiative database; NHANES, National Health and Nutrition Examination Survey; FFQ, Food Frequency Questionnaire; FI, Frailty Index; SOF, Study of Osteoporotic Fracture.

### 3.3 Associations of DII with frailty

**Figure 2:**
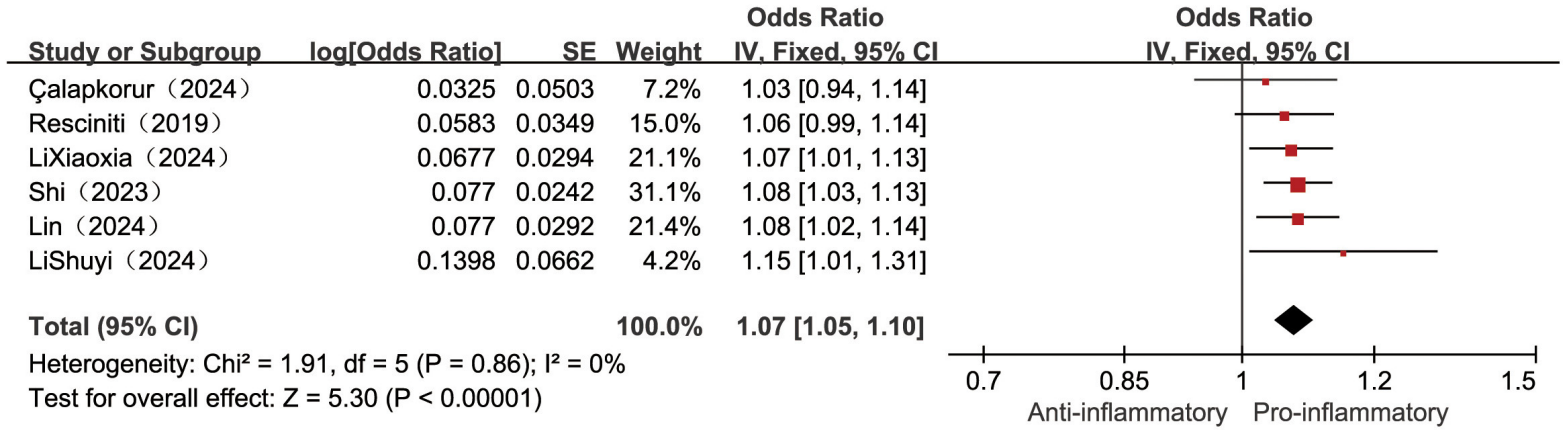
Forest plot of the relationship between the continuous variable DII and frailty risk.

Six included studies (23, 25, 32, 36, 37, 40) examined the correlation between continuously measured DII scores and frailty incidence. Low heterogeneity (*I*^2^ = 0%, *P* = 0.86), supporting the use of a fixed-effects model. Pooled estimates showed a statistically significant positive association between higher levels of DII and increased risk of frailty, with a 7% increase in risk of frailty for each point increase in DII [OR = 1.07, 95% CI (1.05, 1.10), *P* < 0.001, see Figure 2].

**Figure 3:**
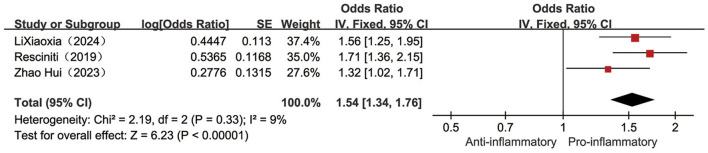
Forest plot of the relationship between the categorical variable DII and the risk of pre-frailty.

Three (25, 29, 36) studies reported the relationship between the categorical variable form DII and the occurrence of pre-frailty conditions, with low heterogeneity across studies (*I*^2^ = 9%, *P* = 0.33), so fixed-effects meta-analysis revealed a 54% higher risk of pre-frailty conditions in the highest category of DII individuals compared to the lowest category of DII individuals [OR = 1.54, 95%CI (1.34, 1.76), *P* < 0.001, see Figure 3].

**Figure 4:**
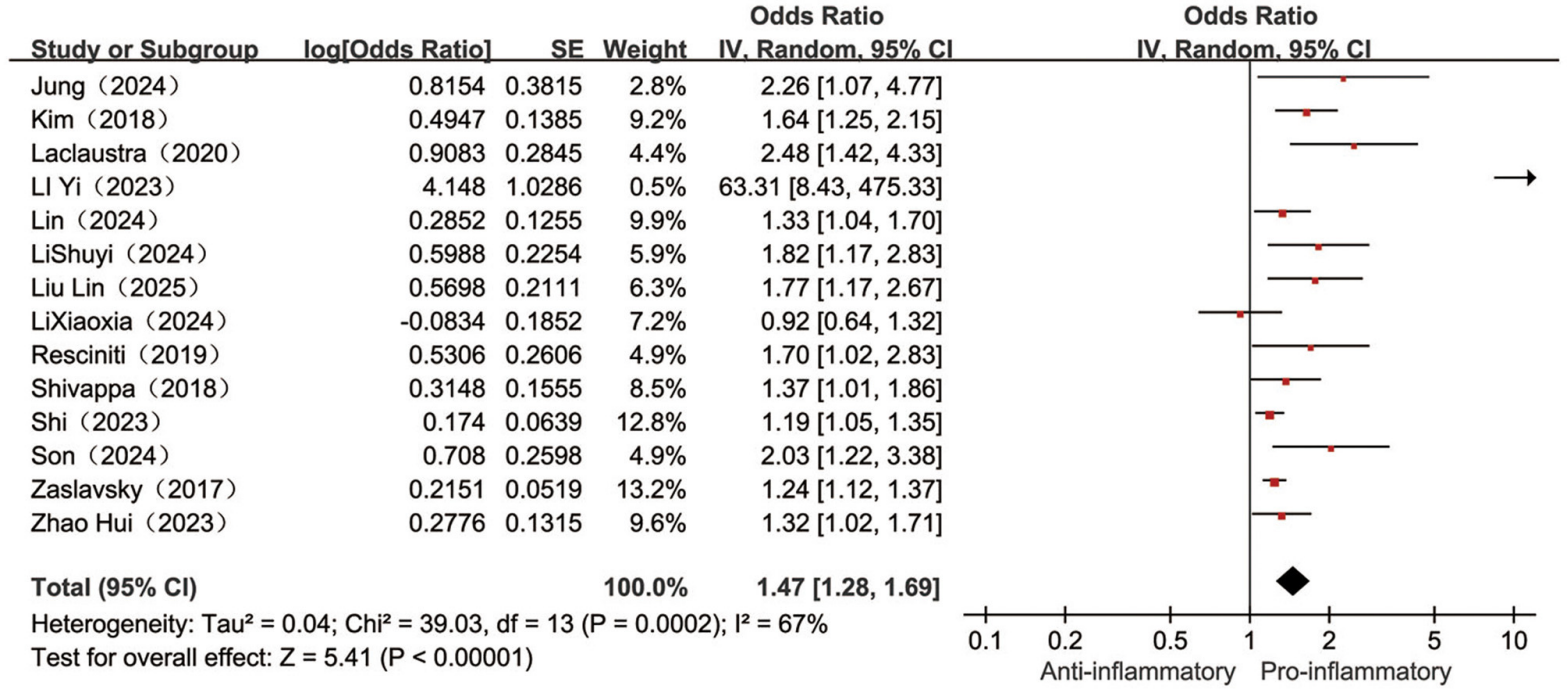
Forest plot of the relationship between categorical variable DII and frailty risk.

Fourteen (23–25, 29–31, 33–40) papers reported on the association between categorical variable form DII and the occurrence of frailty, with a high degree of heterogeneity between studies (*I*^2^ = 67%, *P* < 0.001), meta-analysis with a random-effects model revealed a 47% higher frailty risk in the highest category of DII compared to the lowest category of DII individuals [OR = 1.47, 95%CI (1.28, 1.69), *P* < 0.001, see Figure 4].

#### 3.3.1 Subgroup analysis

Further subgroup analyses of geography, type, sample size, dietary assessment tool, and frailty assessment tool were conducted across studies based on study characteristics to investigate sources of potential heterogeneity, and the influence of different study characteristics on the total effect size (Supplementary Figures 1–4). The results showed that the difference between the occurrence of frailty assessed using the FI Frailty Index and the DII [OR = 1.13, 95%CI (0.79, 1.60)] was not statistically significant (*P* > 0.05); after subgroup analyses based on sample sizes of < 200, 200 to 1,000 (I^2^ = 0%, *P* = 0.80), and >1,000 (*I*^2^ = 37%, *P* = 0.12), inter-study heterogeneity was substantially lowered, and these reductions reached statistical significance (*P* < 0.05), suggesting that sample size may be an important source of heterogeneity, and Table 2 presents the detailed findings from our subgroup analyses.

**Table 2:** Subgroup meta-analysis of the association between DII and frailty risk.

**Subgroup**	**No. of studies**	**OR (95%CI)**	**P-values**	**Heterogeneity**	
				**P-values for within groups**	*I*^2^ **(%)**
**Region**					
Asia	9	1.56 (1.23, 1.97)	0.001	<0.001	75
America	4	1.27 (1.17, 1.39)	<0.001	0.61	0
Europe	1	2.48 (1.42, 4.33)	0.001	—	—
**Study design**					
Cross-sectional	8	1.41 (1.14, 1.76)	0.002	<0.001	73
Cohort	5	1.59 (1.24, 2.04)	<0.001	0.02	64
Case–control	1	1.77 (1.17, 2.67)	0.007	—	—
**Sample size**					
<200	1	63.31 (8.43, 475.33)	<0.001	—	—
200–1,000	4	1.76 (1.45, 2.15)	<0.001	0.80	0
>1,000	9	1.25 (1.17, 1.34)	<0.001	0.12	37
**Methods for dietary assessment**					
FFQ	9	1.38 (1.17, 1.63)	<0.001	<0.001	71
Dietary records	5	1.64 (1.33, 2.01)	<0.001	0.25	26
**Methods for frailty assessment**					
Fried	9	1.78 (1.37, 2.31)	<0.001	<0.001	74
FI	2	1.13 (0.79, 1.60)	0.5	0.11	60
Searle	2	1.22 (1.09, 1.36)	0.005	0.43	0
SOF	1	1.37 (1.01, 1.86)	0.04	—	—

#### 3.3.2 Meta-regression

Meta-regression analysis was used to further explore potential sources of heterogeneity across subgroup factors, incorporating the results of subgroup analyses and the geographic location, type, sample size, dietary assessment tool and frailty assessment tool of each study into the regression equation. The results showed that sample size may be the main source of heterogeneity among the subgroups, as shown in Table 3.

**Table 3:** Meta-regression analysis of DII and risk of occurrence of frailty.

**Variable**	**β**	**SE**	**Z**	**P值**	**95%CI**
**Region (reference group: Europe)**					
America	−0.198	0.468	−0.420	0.700	[−1.67, 0.69]
Asia	0.175	0.646	0.270	0.804	[−1.88, 2.23]
**Sample size (reference group: <200)**					
200–1,000	−4.065	1.106	−3.680	0.035	[−7.58, −0.55]
>1,000	−4.114	1.083	−3.800	0.032	[−7.56, −0.67]
**Study design (reference group: Case–control)**					
Cohort	0.078	0.319	0.250	0.822	[−0.94, 1.09]
Cross-sectional	−0.486	0.458	−1.060	0.367	[−1.95, 0.97]
**Methods for dietary assessment (reference group: dietary records)**					
FFQ	−0.484	0.368	−1.310	0.280	[−1.66, 0.69]
**Methods for frailty assessment (reference group: SOF)**					
Fried	−0.089	0.224	−0.400	0.718	[−0.80, 0.62]
FI	0.012	0.402	0.030	0.977	[−1.27, 1.29]
Seale	0.051	0.332	0.150	0.888	[−1.01, 1.11]

### 3.4 Publication bias

**Figure 5:**
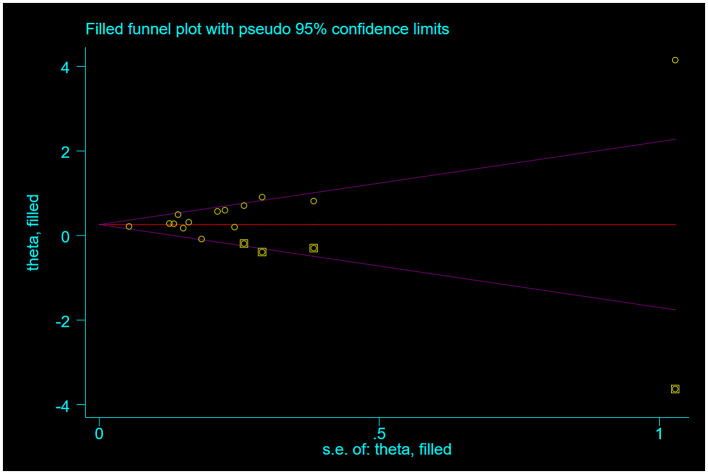
Post-fill funnel plot.

The funnel plot analysis evaluating the association between categorical DII and frailty incidence demonstrated visually detectable asymmetry upon systematic examination, continuing to show statistical significance using the Begg test for publication bias (*P* = 0.016), and Egger's test for publication bias (*P* = 0.007), which suggests the presence of publication bias. Therefore, correction was performed using the trim-and-fill method, and four similar studies were added after three iterations, and the effect sizes were not reversed after the merger [OR = 1.33, 95% CI (1.13, 1.58), *P* = 0.001, see Figure 5].

### 3.5 Sensitivity analysis

**Figure 6:**
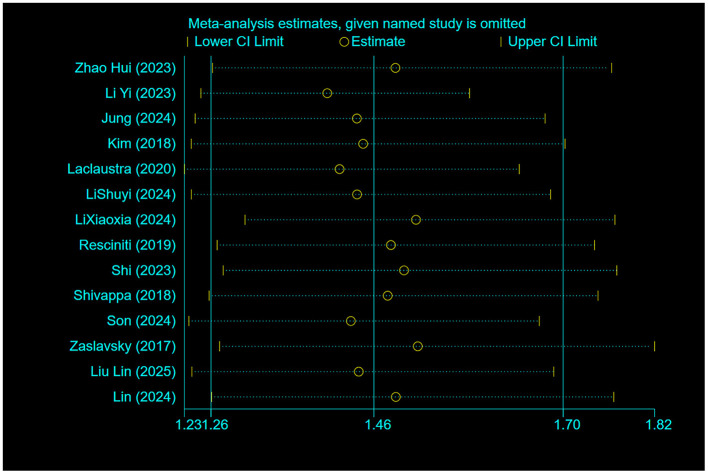
Sensitivity analysis chart.

Sensitivity analyses using the drop-by-drop method as shown in the Figure 6 showed no significant changes in the combined effect sizes, which demonstrates the robustness and reliability of the study outcomes.

## 4 Discussion

Current evidence regarding the correlation between Dietary Inflammatory Index and frailty remains limited and inconsistent. Our study represents, to our knowledge, the most up-to-date systematic review and meta-analysis evaluating the influence of high DII on frailty risk. A meta-analysis of 15 papers on the correlation between DII and frailty with combined effect size ORs found that DII was positively correlated with both frailty and pre-frailty. Sensitivity analyses validated the robustness of our research findings, as no individual study exerted disproportionate influence on the overall results. Results remained consistent in both magnitude and statistical significance after implementing trim-and-fill correction for publication bias. Subgroup analyses demonstrated consistent positive associations between DII and frailty across multiple subgroups, including geographical locations (Asia, America, Europe), study designs (cross-sectional, cohort, case-control), sample sizes (< 200, 200–1,000, >1,000 participants), dietary assessment methods (FFQ, dietary recalls), and frailty measurement instruments (Fried criteria, Searle scale, SOF index). However, studies utilizing the frailty index (FI) showed no significant correlation.

### 4.1 Relationship between DII and risk of pre-frailty in middle-aged and older adults

High-level DII increases the risk of pre-frailty in middle-aged and older adults, which is consistent with the previous meta-analysis results of Donya et al. on the correlation between adherence to the Mediterranean diet and pre-frailty (41). However, in a European community of residents over 70 years old adhering to the Mediterranean diet, no beneficial results of the dietary pattern were found, and the levels of inflammatory markers in the body did not change after a 3-year follow-up (42). The differences in the results of the above studies may be related to differences in the geographic and cultural backgrounds, dietary habits and study designs of the included populations, in addition to differences in the studies' adjustments for confounders that may have contributed to the differences in results. Given the relative paucity of studies exploring the effects of DII on preexisting frailty, more extensive future investigations in diverse populations are needed to fully compare the correlation between DII and the risk of developing preexisting frailty.

### 4.2 Relationship between DII and risk of frailty in middle-aged and older adults

Dietary patterns promoting inflammation (elevated DII) may predispose older individuals to frailty development, in harmony with the results of previous studies (15, 43, 44). American scholars Jayanama et al. (15) explored the correlation between diet and frailty using several different dietary quality scores, such as the Nutritional Index, Healthy Eating Index, and Mediterranean Diet Score, and found that higher scores across all dietary evaluation tools consistently correlated with greater frailty likelihood; in addition, Italian scholars Dominguez et al. (43) included 11 studies and Guan Junyi et al. (44) included 9 studies in their meta-analysis and found that adherence to the Mediterranean dietary pattern (anti-inflammatory diet) was correlated with a decreased risk of frailty.

The underlying etiology of frailty is the chronic inflammatory response that occurs in the body, and food intake provides the body with the energy and substrates needed to cope with this response, so dietary modification based on dietary inflammation to intervene in the inflammatory response has become an important breakthrough point in the effective prevention and treatment of frailty (45). Diets with high DII levels, i.e., pro-inflammatory diets, include red meat, refined grains, fried and barbecued foods, and foods rich in unsaturated fatty acid (46), which contain certain components that can stimulate the body to cause elevated levels of inflammatory factors affecting protein synthesis and metabolism, resulting in a decrease in muscle mass and quality and thus leading to the development of frailty (14, 47). A low DII level diet, or anti-inflammatory diet, is centered on increasing the omega-3 fatty acid (deep-sea fish, flaxseed), dietary fiber (whole grains, vegetables), polyphenol (berries, green tea), and monounsaturated fatty acid (olive oil, nuts) components, while limiting processed meats, refined sugars, trans fats (e.g., fried foods), and high GI carbohydrate intake (48), to increase the body's antioxidant property components, and maintain the stabilization of the intestinal flora, synergistically reversing or delaying the severity of the weakness and its progression (45, 49, 50). Therefore, in the future, anti-inflammatory diets can be promoted for middle-aged and older community members, emphasizing the “diet-inflammation-frailty” chain, popularizing knowledge of dietary inflammation, and conducting regular dietary inflammation screenings to identify people at high risk of frailty for older intervention.

### 4.3 Subgroup discussions

Subgroup analyses were conducted to investigate potential sources of heterogeneity in the DII-frailty association. Subgroup analyses based on sample size showed that heterogeneity among subgroups was within acceptable limits, suggesting that differences in sample size may be the main source of heterogeneity. In addition, other subgroup analyses based on different study geographies, study types, and dietary assessment tools, each with varying degrees of reduced between-study heterogeneity, revealed similar results regarding the relationship between pro-inflammatory diets and the occurrence of frailty, with high levels of DII increasing the risk of frailty in middle-aged and older adults.

Notably the results of subgroup analyses by geography suggested a stronger correlation between DII and risk of frailty in Asian populations compared to the Americas, analyzing the reasons for this may be that the Americas population is chronically adapted to a high glycemic, highly processed diet, which is partially tolerated through intestinal flora adjustments or down-regulation of inflammatory pathways, and that Asian populations are more sensitive to added inflammatory stimuli, while the Asian populations have a relatively low muscle mass, resulting in a unit of The damage to muscle metabolism from inflammatory load may be more pronounced in Asian populations.

When stratified by assessment methodology, studies utilizing the Fried frailty criteria demonstrated stronger, statistically significant correlations with DII scores compared to those employing the Frailty Index (FI), where associations were attenuated and non-significant. The reason for this analysis may be the inconsistency in the criteria for judging frailty, with the Fried frailty phenotype focusing on physical performance such as body weight, muscle strength, and gait speed (51), whereas the FI rating of frailty emphasizes the number of cumulative physical deficits (52), leading to differences in the prevalence and classification of frailty causing differences in the results of the study. Future studies should work on the standardization of frailty assessment tools, including the clear definition of core indicators, the unification of thresholds and the optimization of operational procedures, in order to improve the accuracy and comparability of screening tools, while reducing selection bias caused by different measurement methods.

### 4.4 Strengths

This investigation possesses several notable strengths. Primarily, it represents the most current systematic evaluation and quantitative synthesis examining DII-frailty associations in aging populations, thereby extending previous meta-analytic findings. Our results substantiate emerging evidence that anti-inflammatory dietary patterns may mitigate frailty risk among middle-aged and older adults. Notably, we included studies with inconsistent views for statistical analysis, including more frailty cases and participants than in previous meta-analyses (53), while the inclusion of prospective cohort studies vs. the type of case-control studies increased the reliability of the findings. Secondly we conducted subgroup analyses based on various confounders to elucidate the sources of differences between the included studies. In addition, publication bias was corrected for the presence of studies using the cut-and-patch method, and the results further confirmed the robustness and reliability of the findings.

### 4.5 Limitations

Several limitations should be acknowledged in this investigation. Primarily, the retained research articles were observational, and inferences about causality between the two were not as strong as in interventional studies; second, the calculation of DII relied on self-administered FFQs and dietary recalls, and thus the results may be subject to recall bias; and, although the included studies were all corrected for confounders, they varied with respect to ethnicity, BMI, physical activity, energy intake, and other factors were not identical, and thus bias may have arisen.

## 5 Conclusion

Pooled effect estimates indicate that individuals with pro-inflammatory diets (high DII) exhibit higher odds of developing frailty relative to those consuming anti-inflammatory diets (low DII). The results of this study offer potential improvements in dietary choices for middle-aged and older adults, and at the same time, it provides more research ideas for the prevention of frailty, the existing dietary interventions for frailty are more integrated with other measures, and there are fewer interventional studies on the application of DII in clinical practice. More large-sample, multi-center randomized controlled trials should be carried out to clarify the causal relationship between the two and their intensity. DII helps to carry out scientific research from the source of the disease and provides an innovative strategy for addressing frailty through early detection and intervention. In the future, clinics may consider using the DII for frailty risk assessment and individualized interventions in conjunction with anti-inflammatory dietary recommendations; policymakers may promote the use of the DII in public health guidelines, community nutrition programs, and food policies, and support further research to optimize intervention protocols and assess the potential of DII-based dietary interventions to reduce healthcare expenditures.

## Supplementary material

Figure S1 Forest plot analyzed by regional subgroups.

Figure S2 Forest maps analyzed by subgroups of study types.

Figure S3 Forest plots analyzed by sample size subgroups.

Figure S4 Forest plot analyzed by dietary assessment tool subgroups.

Figure S5 Forest plot analyzed by subgroup of the frailty assessment tool.
