# Supplementary figures and images for: Association of the dietary inflammation index with frailty in middle-aged and older adults: a systematic review and meta-analysis
Source: Front Nutr. 2025 Jul 2;12:1607110. doi: 10.3389/fnut.2025.1607110 (PMC12263374; doi:10.3389/fnut.2025.1607110)

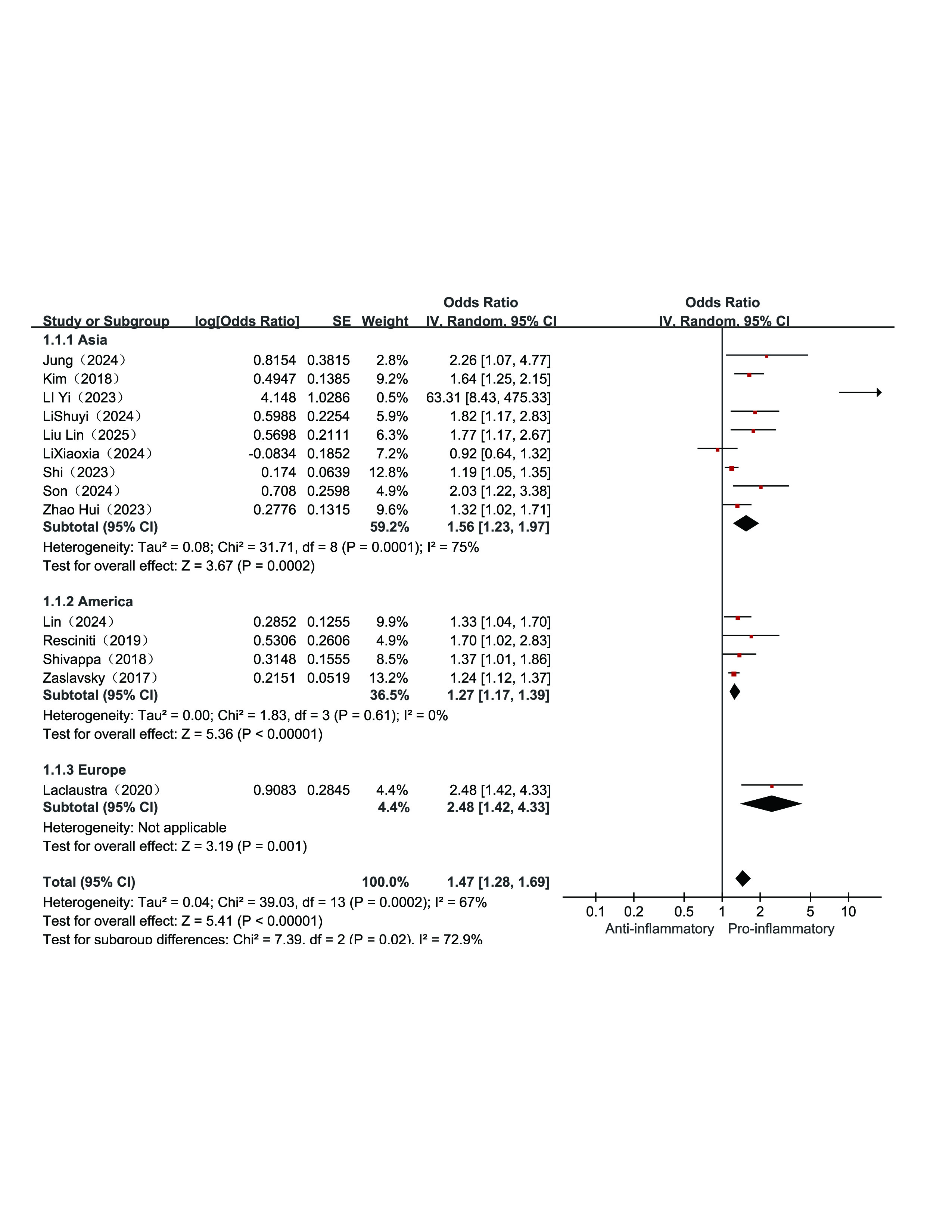

Supplement: Figure S1 — Forest plot analyzed by regional subgroups. [file Data_Sheet_1.zip › Supplementary Figure 1. Forest plot analyzed by regional subgroups.jpg]

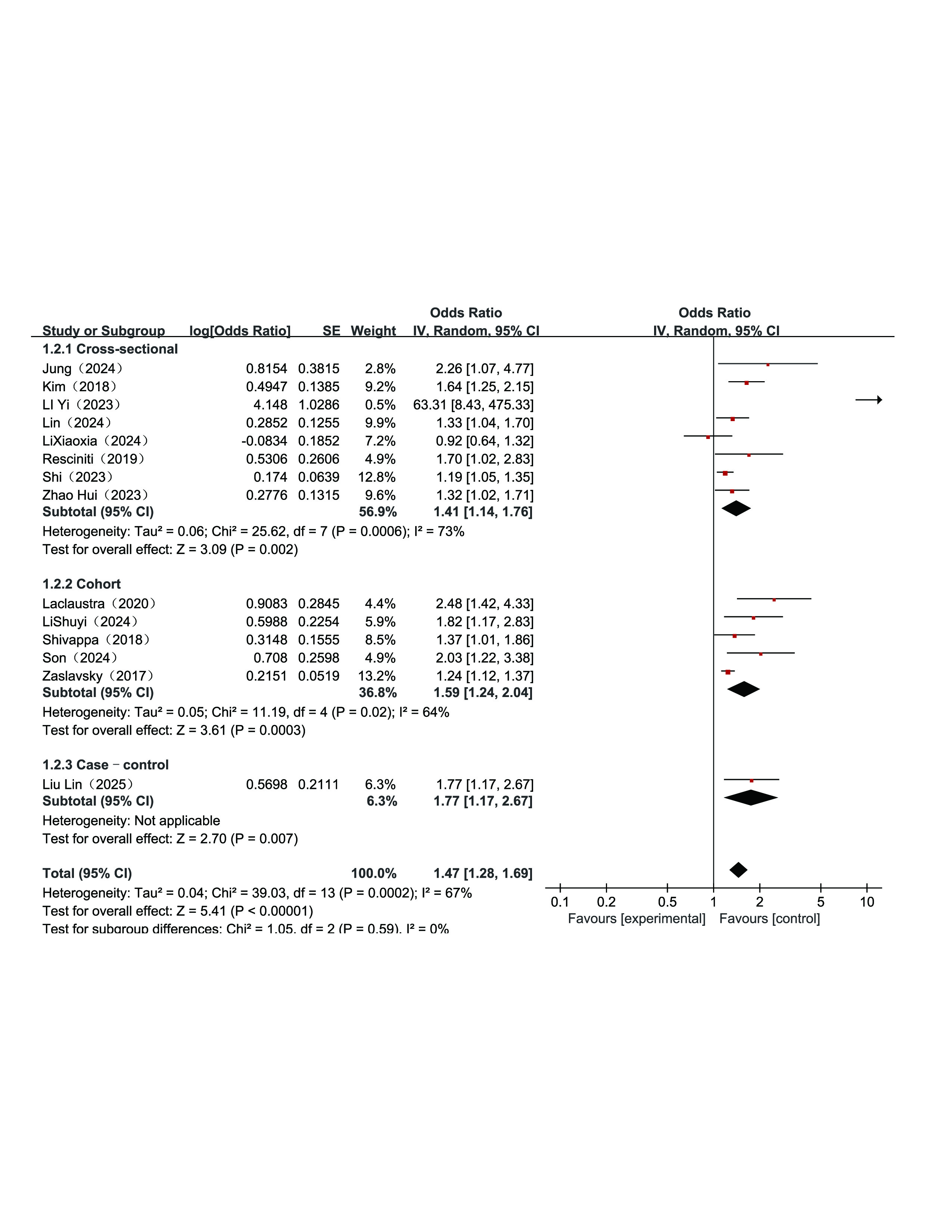

Supplement: Figure S1 — Forest plot analyzed by regional subgroups. [file Data_Sheet_1.zip › Supplementary Figure 2. Forest maps analyzed by subgroups of study types .jpg]

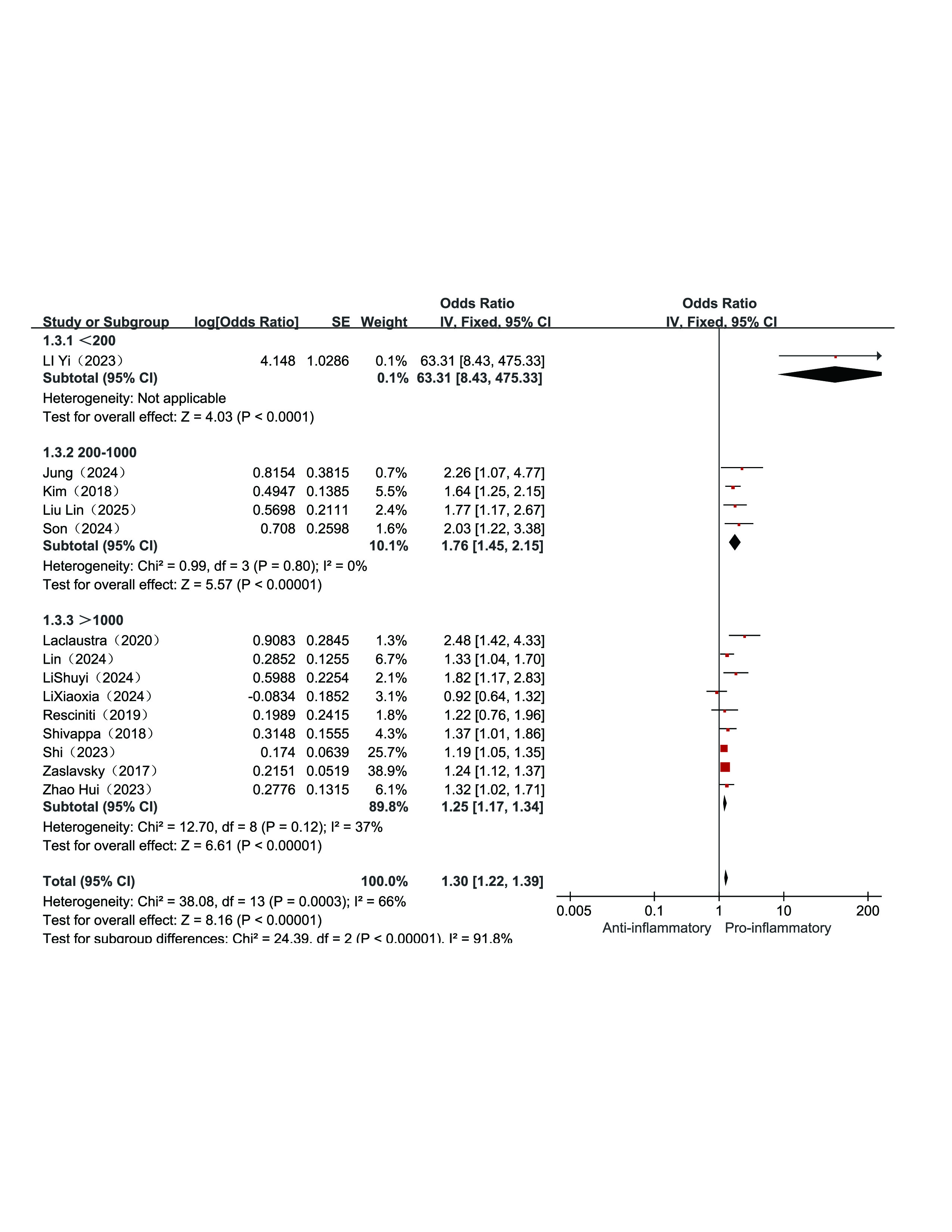

Supplement: Figure S1 — Forest plot analyzed by regional subgroups. [file Data_Sheet_1.zip › Supplementary Figure 3. Forest plots analyzed by sample size subgroups.jpg]

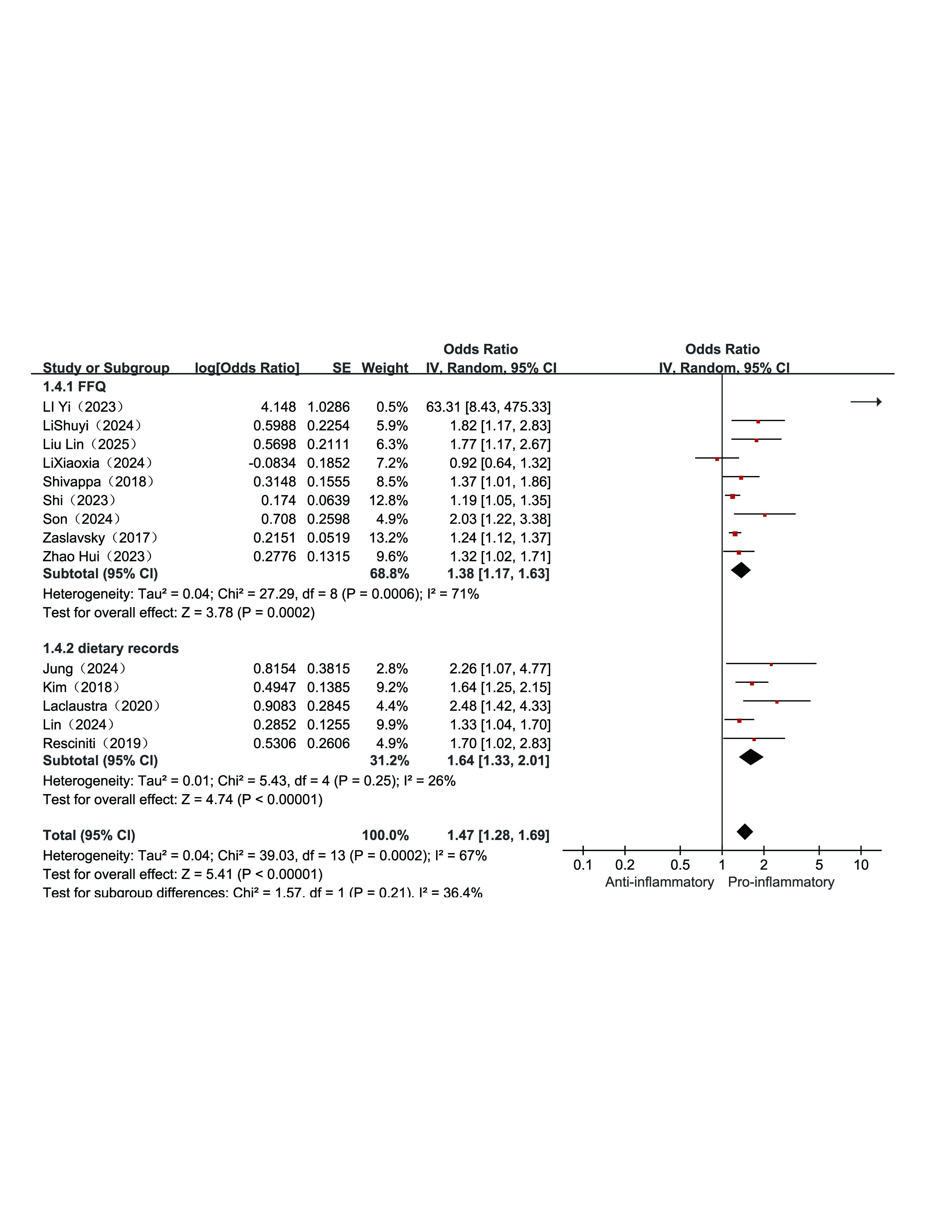

Supplement: Figure S1 — Forest plot analyzed by regional subgroups. [file Data_Sheet_1.zip › Supplementary Figure 4. Forest plot analyzed by dietary assessment tool subgroups.jpg]

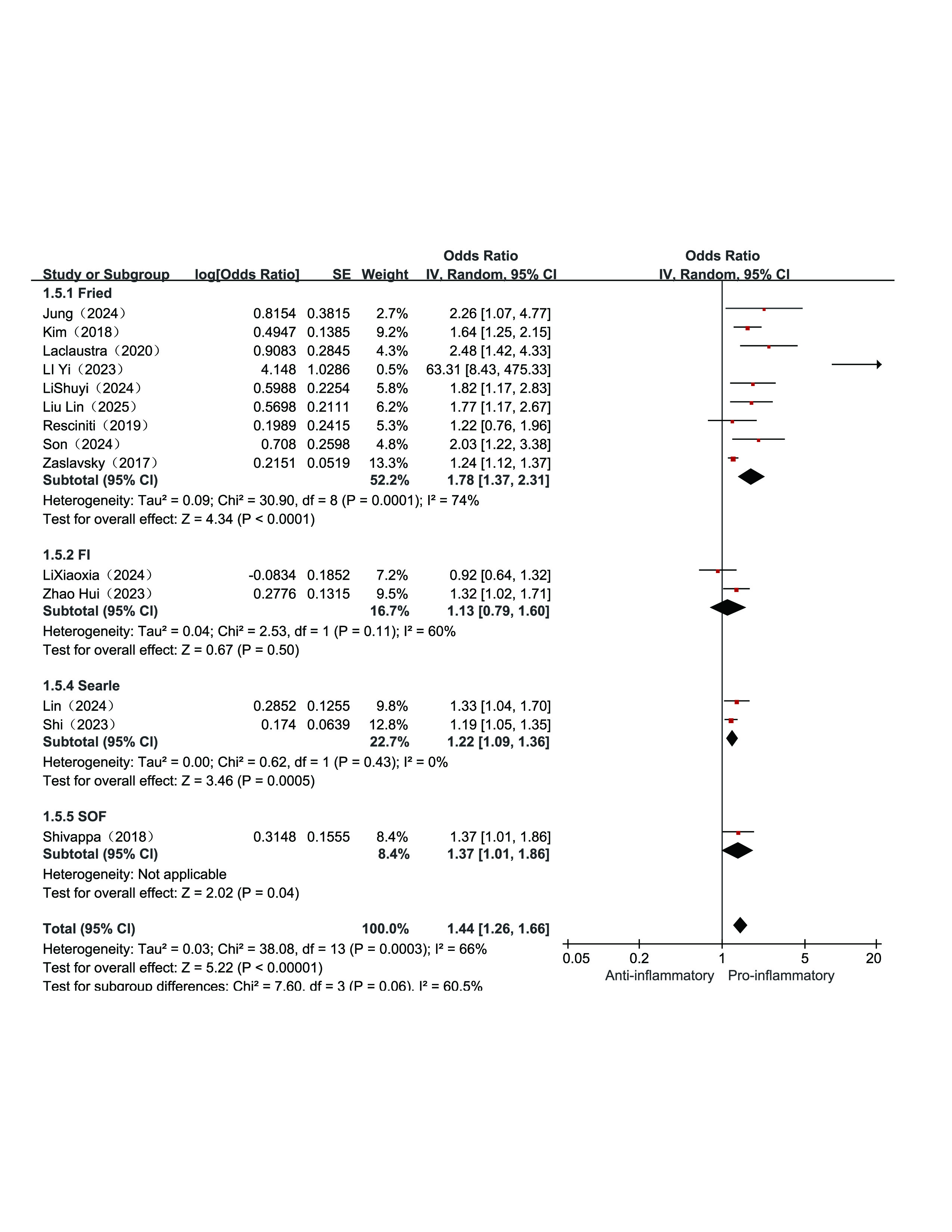

Supplement: Figure S1 — Forest plot analyzed by regional subgroups. [file Data_Sheet_1.zip › Supplementary Figure 5. Forest plot analyzed by subgroup of the frailty assessment tool .jpg]
